# Diet Supplementation with Pomegranate Fruit Alters Distal Gut Microbiota of Healthy Female College Students

**DOI:** 10.3390/microorganisms13020305

**Published:** 2025-01-30

**Authors:** Brant Bandow, Entsar S. Shaaban, Sumudu Rajakaruna, Zeinab Saleh, Sahar A. Abdelaziz, Laila Hussein, Oleg Paliy

**Affiliations:** 1 Department of Biochemistry and Molecular Biology, Boonshoft School of Medicine, Wright State University, Dayton, OH 45435, USA; 2 Department of Home and Economics, Women’s College, Ain Shams University, Cairo 11566, Egypt; 3 Department of Nutrition and Food Sciences, National Research Center, Giza 12622, Egypt

**Keywords:** diet, nutrition, pomegranate, prebiotics, microbiota, gut health

## Abstract

Pomegranate is a fruit that grows abundantly in the Middle East and Africa. It is rich in polyphenols, sugars, fiber, and vitamins, and has long been associated in traditional and alternative medicine with numerous health benefits, including the treatment of diarrhea and gut inflammation. We assessed how regular daily intake of fresh pomegranate can affect the distal gut microbiota of young healthy female students in Egypt, a region with abundant pomegranate production and frequent occurrence of gut dysbiosis. Interrogation of microbiota structure based on the sequencing of the 16S ribosomal RNA gene amplicons indicated that subject-to-subject variability was the main driver of microbiota community differences. Nevertheless, pomegranate consumption led to changes in the abundances of several genera including increased levels of *Saccharofermentans*, *Enterococcus*, and *Prevotella*. The relative counts of *Dysosmobacter*, *Coprococcus*, and *Collinsella* decreased after pomegranate intake. The magnitude of community structure shift after diet intervention correlated with the increase in the total polyphenol concentration measured in subjects’ urine. The overall ratio of presumed beneficial-to-detrimental microbes was also improved with pomegranate addition to the diet, supporting the advantageous effects of pomegranate eating.

## 1. Introduction

**Figure 1:**
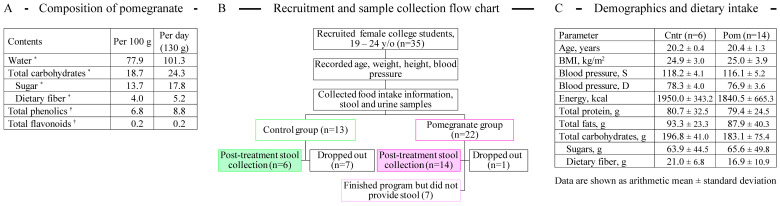
(**A**) Typical composition of fresh pomegranate arils. * Data are from the USDA National Nutrient Database [22]; ^†^ Data are from ref. [23]. (**B**) Recruitment and sample collection flow chart. (**C**) Demographics and dietary intake of profiled subjects at the baseline. Data are shown as arithmetic mean ± standard deviation.

Many vegetables and fruits provide not only basic nutritional carbohydrates, lipids, and proteins, but also contain a large variety of bioactive compounds [1]. Many fruits are especially rich in polyphenolic metabolites including flavonoids and tannins, which possess potent antioxidant and anti-inflammatory properties [2]. Among fruits, the arils and juice of pomegranate (*Punica granatum* L.) have one of the highest polyphenolic contents, with levels higher than those found in the red wine and green tea [3] (Figure 1A). The fruit arils, with characteristic ruby color, make up approximately 33% to 40% of the fruit weight and are the richest natural source of bioactive components that include ellagic acid, ellagitannins, punicic acid, and other fatty acids, flavonoids, estrogenic flavonols, and flavones. In addition to these compounds, pomegranate fruit is an abundant source of sugars (primarily glucose and fructose), dietary fiber, vitamins C, E, and K, potassium, folate, and anthocyanin pigments [4].

Pomegranate fruit and juice have many health benefits [5]. The presence of many polyphenols such as anthocyanins, ellagitannins, and ellagic acid provides strong antioxidant and reactive oxygen species scavenging capabilities, which in turn leads to an anti-inflammatory effect [3,6]. For example, dietary supplementation of pomegranate extract to mice maintained on high-fat, high-sugar diet reduced colitis and lowered inflammatory markers [7]. Pomegranate extract is able to inhibit the activation of pro-inflammatory master regulator NF-κB and diminish the development of type 2 diabetes [8], and consumption of pomegranate juice for six weeks decreased lipid peroxidation in subjects with metabolic syndrome [9]. Pomegranate consumption can decrease the level of lipids in the blood as well as the overall triglyceride content: a three-week dietary intervention with pomegranate juice significantly decreased the serum levels of lipids and cholesterol, with concomitant rise in the body phenol pools [10,11]. Finally, pomegranate has also shown promise in improving the outcomes of cancer treatments (reviewed in [12]).

In addition to the direct effects on human physiology described above, pomegranate has also been found to have prebiotic properties supporting the growth of beneficial gut microbes. Li et al. showed that pomegranate and pomegranate juice promoted the growth of beneficial *Bifidobacterium* and *Lactobacillus* bacteria, at the same time inhibiting members of *Enterobacteriaceae*, which are known to contain many pathogenic species [13]. Furthermore, pomegranate peel exhibited antimicrobial action when tested against human-associated bacteria including *Escherichia coli*, *Pseudomonas aeruginosa*, and methicillin-resistant *Staphylococcus aureus* [14]. The consumption of pomegranate juice can also stimulate the production of beneficial short chain fatty acids in the gut lumen [15]. In addition, gut microbiota is vital to achieve the health benefits of pomegranate polyphenols, since microbial enzymes convert pomegranate flavonoids such as ellagitannins into urolithins that are then absorbed into the circulation [16]. Some strains of *Lactobacillus* are also able to degrade hydrolyzable tannins to the antioxidant derivatives gallic acid and pyrogallol [17].

Pomegranate is a fruit that grows abundantly in the Middle East and Africa, including Egypt. Cases of gut dysbiosis are reported frequently in the Egyptian population [18], and the burden of intestinal infections is high [19]. Such vulnerability to intestinal infections and dysbiotic gut environment has been linked previously to the level of hygiene and the integrity of intestinal barrier function [20,21]. Owing to the many gut-health-promoting effects of pomegranate and the availability of this fruit in the Middle East region, in this study we aimed to assess the beneficial effects of daily intake of fresh pomegranate arils on the gut microbiota structure among Egyptian female college students. Our hypothesis was that the consumption of fresh pomegranate will shift subjects’ gut microbiota towards a more beneficial state.

## 2. Materials and Methods

### 2.1. Study Cohort

This study was a randomized controlled trial, and 35 Egyptian female students were recruited from the Women’s College—Ain Shams University in Cairo, Egypt. The study was carried out in accordance with the guidelines of the Human Subjects Protection Committee of the National Research Center (NRC) in Giza, Egypt, and the protocol #422/16 was approved by the Medical Ethical Committee of the NRC. All participants gave written informed consent before the study began. The inclusion criteria for participation were being of age 18–30, no non-declared or known pathology, and not having taken antibiotics, medication, or pre/probiotics in the two months before the study. The exclusion criteria were age outside the inclusion range, history of diabetes, hypertension, heart disease, or endocrine disorders, current pregnancy or lactation, and known allergy or hypersensitivity to any food.

A total of 27 students completed the 3-week pomegranate supplementation trial (see recruitment flow chart in Figure 1B). The trial was a randomized controlled two-arm study, and the participants were randomized into either a pomegranate or control group. The intervention consisted of a daily intake of 130 g of peeled pomegranate arils for three weeks; the control group consumed their regular diet with no additional supplements. Pomegranate fruits were purchased in bulk from the Obour public market in Cairo, Egypt. The pomegranate fruits were peeled manually, and the red arils were distributed in aliquots of 130 g in airtight polyethylene bags protected from light and saved in the refrigerator for a maximum of one week. The intake of pomegranate was well accepted with no adverse effects. Participants were instructed to maintain their regular diet and not to consume polyphenol rich fruits throughout the study period. Participants were asked to complete a 3-day dietary record before and after the trial, and the compiled dietary data are provided in Figure 1C. No significant differences were detected in the energy, carbohydrate, protein, and fat intake between the baseline and the post-study time period.

### 2.2. Data and Sample Collection

Demographic factors were assessed via a structured questionnaire. At the baseline, a standard questionnaire concerning date of birth, education, smoking habit, health-related information history, and medications were obtained via face-to-face interviews. All participants had a physical examination including measurement of height and weight. Body mass index (BMI) was calculated as weight (kg) divided by squared height in meters (m^2^). A subset of volunteers (6 in control group and 14 in the pomegranate group, see Figure 1C) collected their fecal and urine samples before (baseline; day 0) and after (post-trial; day 22) the intervention period into the provided containers. The fresh fecal samples were homogenized immediately after collection and were frozen within 2 h after defecation. The urine samples were frozen upon receipt until further analyses.

### 2.3. Isolation of Genomic DNA and High-Throughput DNA Sequencing

Total genomic DNA (gDNA) was isolated from approximately 150 mg of fecal material using ZR Fecal DNA Isolation kit (Zymo Research Corporation, Irvine, CA, USA), as we performed previously [24]. Due to the shipment of the fecal material from Egypt to the United States, several samples became degraded and either could not provide gDNA of sufficient quality, or significant artifacts were detected in their sequencing data. The final dataset thus consisted of 11 paired samples in the pomegranate group and 6 paired samples in the control group.

For the interrogation of microbial composition, the V1V2 variable region of the 16S ribosomal RNA gene was amplified using the conserved degenerate primers AGRGTTYGATYMTGGCTCAG and GCWGCCWCCCGTAGGWGT. The forward primers contained a 6–7 nucleotide barcode to permit sample pooling. PCR amplifications were performed in a 25 μL volume with 25 ng of gDNA template, and 4 cycles of linear followed by 25 cycles of exponential amplification [25]. Amplicons were equimolarly pooled, and high-throughput sequencing was performed on the Ion Torrent Personal Genome Machine using Ion 318 and 316 chips (Thermo Fisher Scientific, Waltham, MA, USA) following the manufacturer’s protocols. After quality filtering, an average of 14,694 reads was obtained per sample. Low-quality (average Q < 25) and short (<160 nct) reads were removed. High-quality reads were analyzed in QIIME, similarly to our previous approach [26]. Briefly, microbial phylotypes were defined by clustering at 97% sequence similarity. Taxonomic annotation of phylotypes was performed with Ribosomal Database Project Classifier v2.11 against the RDP 16S rRNA training set 19. All taxon counts were divided by the calculated average 16S rRNA gene copy number for that taxon in order to derive cell abundances [27,28], and these cell counts were then rarefied to the same (lowest) number. This final dataset was used for all multivariate analyses.

### 2.4. Prebiotic Index Calculation

We estimated the total counts of presumed “beneficial” and “detrimental” human gut microbes in all samples as we described previously [29]. Total beneficial microbes consisted of combined abundances of *Akkermansia*, *Bifidobacterium*, *Eubacterium*, *Faecalibacterium*, *Lactobacillus*, *Roseburia*, and probiotic *Streptococcus*. Total detrimental microbes combined the abundances of *Clostridioles difficile*, Desulfovibrionaceae, Enterobacteriaceae, *Fusobacterium*, and *Helicobacter*. The data are displayed as the ratio between total beneficial to total detrimental microbial counts (BD ratio).

### 2.5. Measurements of Urinary Polyphenols and Creatinine

The measurement of total polyphenolic compounds (PPs, include gut microbiota produced urolithins) in subjects’ urine followed the method of Roura et al. [30]. Briefly, centrifuged urine samples (1 mL) were acidified with hydrochloric acid (17 µL), and 500 µL aliquots were processed for the colorimetric determination of the PPs by the addition of Folin–Ciocalteu reagent (50 µL), followed by 20% sodium carbonate (600 µL). Aliquots were left in the dark for 60 min, and the developed absorbance was measured at OD_765nm_ against a blank. A series of standard dilutions of gallic acid (0.215 mg/mL) was processed alongside this to construct a standard curve. The amounts of measured PPs were adjusted to the value of creatinine in each sample and were expressed as grams of total polyphenols per gram of creatinine. Urinary creatinine was determined by the alkaline picrate method [31].

### 2.6. Statistical Data Analyses

Statistical procedures were carried out in R v4.0, SPSS v19, and MATLAB 2015a using approaches that we described earlier [32]. Because the dataset displayed high variability in taxon abundances among samples, a geometric mean was used in place of an arithmetic average to estimate group means. Multivariate ordination algorithms included principal components analysis (PCA), weighted UniFrac distance-based principal coordinates analysis (PCoA) [33], redundancy analysis (RDA), canonical correspondence analysis (CCA), and orthogonal projections to latent structures discriminant analysis (OPLS-DA) [32]. Distributions of abundances of discriminating genera were visualized with boxplots, as we performed previously [34]. PICRUSt v2.0 and STAMP v2.1 software were used to impute and analyze the predicted microbiota community functions as we described [26].

Differentially abundant pathways were defined based on the Welch’s two-sided *t*-test. The statistical significance of differences between the baseline and post-trial cohorts was assessed with the paired samples *t*-test run on the log-transformed dataset in order to better fulfill test assumptions.

## 3. Results

### 3.1. Diet Supplementation with Fresh Pomegranate

We recruited healthy female students into a 21-day diet supplementation with fresh pomegranate (Pom). Another set of randomly chosen female students served as controls (Cntr). Overall, there was no statistical difference between subject groups in age, BMI, blood pressure, and macronutrient and total daily energy ingestion (Figure 1C), though noticeable subject-to-subject variability was observed among participants. No significant differences were detected in the energy, carbohydrate, protein, and fat intake between the baseline and the post-study time period.

**Figure 2:**
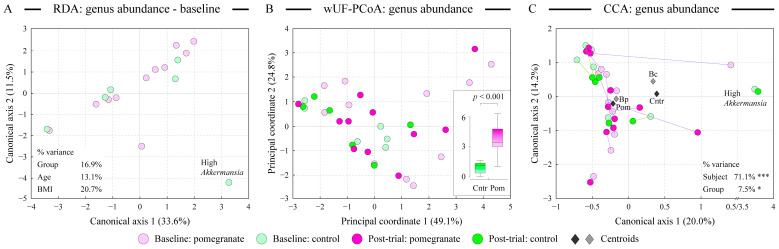
Comparison of distal gut microbiota composition among participating subjects. Panel (**A**) displays the distribution of baseline samples in a constrained ordination space based on the redundancy analysis (RDA) algorithm and shows no statistically significant difference between Pom and Cntr groups in their gut microbial composition before the trial. Subject identity, age, and BMI were used as constrained variables; none were statistically significantly associated (at α = 0.05 level) with microbiota composition. Similarity of microbial communities at the genus taxonomical level among all samples was assessed by unconstrained weighted UniFrac-based principal coordinates analysis (wUF-PCoA), panel (**B**), and by the constrained canonical correspondence analysis (CCA), panel (**C**). Both approaches show high inter-personal variability among subjects, but reveal statistically significant differences between Pom and Cntr groups in their distal gut microbiota composition. The percentage of total dataset variance explained by each axis is shown in parenthesis. The panel (**B**) insert shows the distribution of distances in the PCoA space for the baseline-control (Cntr) and baseline-pomegranate (Pom) sample pairs. Statistical significance was based on the Mann–Whitney U test. CCA plot panel (**C**) displays the position of centroids of each class of categorical explanatory variables (Bc—control group baseline samples; Bp—pomegranate group baseline samples; Cntr—post-trial control group samples; Pom—post-trial pomegranate samples). The positions of each pair of samples (pre/post-trial) in the CCA plot are linked by a line: shorter line represents smaller change in microbiota community structure. Note the break in the CCA X axis scale to facilitate visualization of the outlier sample pair. The relative contribution of explanatory variables to the overall variance of the dataset is shown in the panel (**A**,**C**) inserts; *: *p* < 0.05, ***: *p* < 0.001.

The analysis of fecal microbiota in enrolled subjects prior to the start of the trial indicated that there was no statistically significant difference between Pom and Cntr groups in their gut microbial composition (Figure 2A). Similarly, age and BMI were not statistically significantly associated with microbiota composition. Subject-to-subject variability was generally high. For example, one subject’s fecal microbiota was distinctly different from the rest of the samples due to the presence of an unusually high abundance of genus *Akkermansia* (see Figure 2A).

### 3.2. Daily Consumption of Fresh Pomegranate Alters Distal Gut Microbiota

Phylogenetic distance-based PCoA ordination analysis was used to compare the similarity of fecal microbiota in all baseline and post-trial samples (Figure 2B). Constrained canonical correspondence analysis (CCA) was utilized to statistically assess the influence of categorical explanatory variables on the measured microbiota profiles (Figure 2C). Samples did not cluster in either ordination space according to the group identity, and inter-subject differences were the primary driver of microbiota community dissimilarity among samples (71.1% of overall explained variance in CCA, *p* < 0.001). Group identity (Pom vs. Cntr) was weakly, but still statistically significantly, associated with the fecal microbiota composition (*p* < 0.05, see Figure 2C). Interestingly, the daily intake of fresh pomegranate arils altered the baseline microbiota of subjects significantly more than that observed in the control group, as was evident from the distribution of baseline-vs-post-trial distances in the PCoA space (*p* < 0.001, see Figure 2B insert). We previously revealed a similar trend of larger prebiotic-associated shifts in fecal microbiota in young Egyptian adults provided with the fermented sour sobya [34]. The alterations of baseline microbiota were dependent on the subject, as can be assessed by the varied length of baseline to post-trial connecting lines shown in Figure 2C. Based on the CCA distances, two subjects in the pomegranate group had a large change in their microbiota structure and five showed medium change, whereas the remaining four subjects in the same group revealed minor microbiota changes. The magnitude of these alterations might depend on the ability of the subject’s gut microbiota to metabolize pomegranate polyphenols, as was shown previously ([35] and see below). In contrast, no subjects in the control group showed large microbiota alterations, with three out of six subjects only displaying minor deviations from the baseline.

### 3.3. Abundant Taxa of Egyptian Gut Microbiota

**Figure 3:**
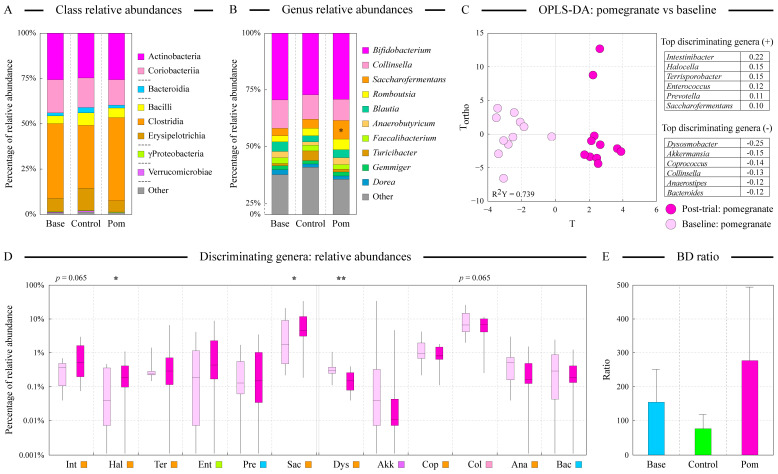
Differences in class and genus abundances among sample groups. The relative abundances (geometric mean within each group) of the top eight classes and top ten genera were comparable among sample groups, as shown in the stacked column charts visualized in panels (**A**,**B**), respectively. Baseline group combines samples from the baseline control and baseline pomegranate samples. Classes are ordered based on their phylum assignment. Note that due to the mathematical nature of geometric mean calculation, relative abundances are not directly comparable between class and genus abundance distributions. Star denotes the statistically significant difference (at α = 0.05 level) in the *Saccharofermentans* abundance among sample groups, as calculated by the analysis of variance algorithm. Note the compression of the Y axis between 0% and 25% relative abundance values in panel (**B**). The results of the orthogonal projections to latent structures discriminant analysis (OPLS-DA) of the genus abundance dataset between pomegranate baseline and post-trial groups are displayed in panel (**C**). The top discriminating genera (with at least 0.5% relative average abundance in at least one group) are shown in the tables. “+” genera increased in post-trial samples; “−” genera decreased; values represent the weights of each genus in the PLS model. R^2^Y denotes the amount of between-group variation explained by the model. The distributions of abundances of these discriminating genera among samples of each group are depicted in panel (**D**) box and whiskers plots; the whiskers indicate the range of minimum and maximum values. Note the logarithmic scale of Y axis. Class assignment is shown next to the abbreviated name of each genus following the color scheme presented in panel (**A**). Where shown, single and double stars indicate the statistical significance of taxon abundance difference between two groups (at α = 0.05 and α = 0.01 levels, respectively, based on the paired samples *t*-test). Genus abbreviations are: Int—*Intestinibacter*; Hal—*Halocella*; Ter—*Terrisporobacter*; Ent—*Enterococcus*; Pre—*Prevotella*; Sac—*Saccharofermentans*; Dys—*Dysosmobacter*; Akk—*Akkermansia*; Cop—*Coprococcus*; Col—*Collinsella*; Ana—*Anaerostipes*; Bac—*Bacteroides*. Panel (**E**) visualizes the calculated ratio of beneficial-to-detrimental microbes (BD ratio) for the three groups. Error bars represent the standard errors of the mean of BD ratios within each group.

Figure 3A,B display the relative cell abundances of fecal microbiota at the class and genus taxonomical levels. At the class level, Clostridia, Actinobacteria, and Coriobacteriia dominated. The abundance of class Bacteroidia was low, in contrast with the usually observed high abundance of this class in populations of industrialized countries [36,37]. There were no statistically significant alterations during diet supplementation trial at the class level. At the genus level, the most abundant genera included *Bifidobacterium* (class Actinobacteria), *Collinsella* (class Coriobacteriia), *Saccharofermentans*, *Romboutsia*, and *Blautia* (all from class Clostridia) (Figure 3B). The large abundance of *Bifidobacterium* is notable, since the prevalence of this genus tends to wane during adolescence in industrialized populations [38]. Due to the high subject-to-subject variability in microbiota composition, only the abundance of *Saccharofermentans* differed significantly between the groups among the top ten genera.

### 3.4. Many Genera Contribute to the Observed Alterations of Distal Gut Microbiota After Pomegranate Consumption

We utilized an OPLS discriminant analysis to reveal genera that accounted for the shift in microbiota community structure after pomegranate intake. The generated model could explain 73.9% of the between-class variability (Figure 3C), and the top genera contributing to the separation of pre- and post-pomegranate consumption samples are shown in Figure 3C tables. The distribution of relative abundances of these genera among both sample groups is visualized with boxplots plotted in Figure 3D. The majority of the discriminating genera represented class Clostridia, and we observed noticeable variability in the abundances of each genus among samples, further highlighting inter-subject variability of microbiota composition and its response to pomegranate supplementation.

### 3.5. Pomegranate Supplementation Promotes Beneficial Microbial Communities

We estimated a “healthy” state of each microbial community by summing the abundances of known beneficial and detrimental microbes and calculating the beneficial-to-detrimental BD ratio. As can be observed in Figure 3E, pomegranate supplementation improved this BD ratio by almost 2-fold, though due to the high inter-personal variability, the difference did not reach the level of statistical significance. The change was due to a modest increase in total counts of *Bifidobacterium* combined with the reduction in total Enterobacteriaceae, which is consistent with previous in vitro findings [13]. No such improvement was evident for the control group of subjects.

### 3.6. Alterations in Predicted Microbial Functions upon Pomegranate Consumption

**Figure 4:**
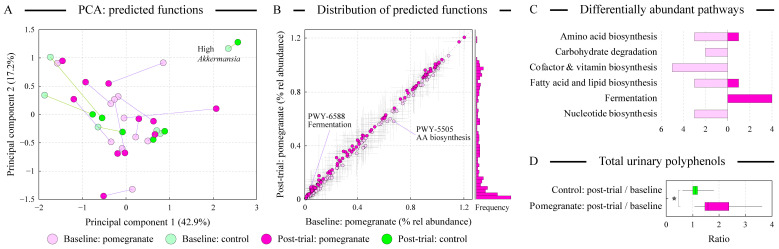
Functional analysis of the distal gut microbiota. Panel (**A**) displays the distribution of samples in an unconstrained principal components analysis (PCA) space based on the abundances of predicted metagenomic functions and reveals high inter-personal variability among subjects. The positions of each pair of samples (pre/post-trial) are linked by a line: shorter line represents smaller change in the microbial functional repertoire. The percentage of total dataset variance explained by each axis is shown in parenthesis. Comparison of the distribution of predicted functional pathways between baseline and post-trial samples in the pomegranate cohort is displayed in panel (**B**). Axes show relative abundance (%) of each pathway in the baseline and post-trial samples. The frequency distribution of these pathways among post-trial samples is shown on the right, highlighting the relatively low presence of the majority of annotated pathways. Thin gray lines depict confidence intervals for the abundance of each pathway. The counts of differentially abundant pathways (minimum 1.5-fold difference) between the baseline and post-trial pomegranate samples are displayed in panel (**C**); only pathway categories with at least two cumulative differentially abundant pathways are shown. The locations of two of these pathways are highlighted on the pathway distribution plot shown in panel B. The distributions of ratios of total polyphenols between each subject’s baseline and post-trial samples are shown in panel (**D**). Star denotes the statistically significantly higher amount (at α = 0.05 level) of urinary polyphenols after pomegranate consumption as measured by the Mann–Whitney U test.

We used PICRUSt2 algorithm to predict the set of microbial functions in each fecal sample [26]. Ordination analysis of this functional dataset showed no clear separation of samples based on the supplementation group (Figure 4A). Nevertheless, similar to the dispersal of samples based on the microbial abundances (see Figure 2B,C), the pomegranate group displayed more frequent shifts in the distribution of microbial functional abundances after the supplementation. The comparative distribution of pathway abundances among pomegranate baseline and post-trial samples is shown in Figure 4B. As expected, the majority of pathways were of low abundance highlighting the previously noted diversity of functional genes in the gut microbial communities [39]. A number of functions differed substantially (at least 1.5-fold) between the baseline and pomegranate samples. Their allocation among pathway categories is displayed in Figure 4C. The main increase in prevalence after pomegranate intake was evident for the pathways in the fermentation category, likely to take advantage of the presence of additional sugars and dietary fiber in pomegranate arils [4]. In contrast, functions in the cofactor and vitamin biosynthesis pathways were often less prevalent after pomegranate supplementation. Pomegranate is known to contain substantial amounts of vitamins and other bioactive compounds, plausibly explaining the reduced need for the encoding of such metabolic functions in the gut microbiome.

### 3.7. The Magnitude of Microbiota Alterations Correlates with the Increase in Urinary Polyphenols

We measured the concentrations of total polyphenols in the urine of each profiled subject before and after the dietary supplementation trial (Figure 4D). There was no change in the PP amounts among the control group (average post-trial/baseline ratio 1.08), whereas the consumption of pomegranate led to a noticeable increase in total urinary PPs (average post-trial/baseline ratio 1.94, statistically significantly different from the control cohort with *p* = 0.029 based on the Mann–Whitney U test). Intriguingly, we also uncovered a statistically significant association (Spearman correlation R_S_ = 0.507, *p* = 0.027, all Pom and Cntr samples) between the baseline to post-trial change in urinary polyphenols and the magnitude of microbiota community structure shift (as represented by the distance in PCoA space between the baseline and post-trial samples, see insert in Figure 2B). Similar stratification of gut microbiota based on their ability to metabolize pomegranate bioactive compounds was noted recently [35,40].

## 4. Discussion and Conclusions

In this study, we determined the response of gut microbiota of young female students to the daily diet supplementation with fresh pomegranate arils. The motivation for the study was, on one hand, a desire to improve gut environment in populations with lower intestinal health such as in Egypt, and on the other hand, our interest in using pomegranate, a fruit rich in polyphenols and dietary fiber, as a potential prebiotic supplement.

The analysis of fecal microbiota structure indicated that inter-subject variability was the main contributor to the overall variance of microbiota abundance dataset, consistent with previous reports [41,42]. An example of such microbiota uniqueness is presented by the pair of pre/post-trial samples from a subject in the control group who was remarkably abundant in the members of genus *Akkermansia* (see Figure 2A,C and Figure 4A). Nevertheless, the contribution of diet supplementation had a statistically significant effect on the microbiota variance (see Figure 2C), and more subjects in the pomegranate group showed substantial shifts in their gut microbial community after the supplementation compared with the controls (see Figure 2B,C and Figure 4A). Intriguingly, the higher magnitude of observed shifts in microbiota structure upon pomegranate consumption was associated with a higher level of total polyphenols in the subject’s urine. This implied that changes in microbiota community upon pomegranate supplementation depended on the community’s ability to release and metabolize pomegranate phenolics. Together, these data indicate that utilization of pomegranate bioactive compounds by gut microbiota varies among subjects, and that gut microbiota regulates the availability of pomegranate polyphenolic compounds (such as urolithins) to the human host [16].

By utilizing an OPLS discriminant analysis, we identified a number of genera that were altered in their abundance in the pomegranate group subjects after the pomegranate intake (see Figure 3C,D). Among these, clostridial genera *Saccharofermentans*, *Intestinibacter*, and *Terrisporobacter*, which all increased after pomegranate consumption, are associated with the anaerobic breakdown of sugars and likely took advantage of the additional presence of glucose and fructose in the pomegranate arils [43,44]. Two other genera with higher abundance after pomegranate ingestion, *Limosilactobacillus* (previously members of *Lactobacillus*) and *Enterococcus*, are lactic acid producing bacteria with previously shown positive health effects [45]. Finally, *Prevotella* and *Halocella*, the numbers of which were also boosted by pomegranate eating, have been shown to be good degraders of dietary fiber [46,47]. In contrast, dietary consumption of pomegranate reduced the abundances of *Collinsella* and *Leuconostoc*, two genera previously linked with gut inflammation and gastrointestinal diseases [48,49]. These increases in health-promoting members of human gut microbiota and reductions in harmful species led to an improvement in the estimated ratio of total beneficial to total detrimental microbes in the post-pomegranate consumption samples (see Figure 3E), supporting the beneficial effect of pomegranate addition to the diet.

Several of our findings agree with the recently published report by Li and colleagues [35]. In the referenced study, 20 healthy adult volunteers of both sexes from the California region of United States received 1000 mg of pomegranate extract daily for four weeks. Subjects could be stratified into urolithin producers and non-producers. Among the former, gut microbiota members of genera *Lactobacillus*, *Enterococcus*, and *Prevotella* increased after taking the pomegranate supplement, whereas the abundance of *Collinsella* decreased in the gut [35], matching our findings in a healthy Egyptian cohort.

To conclude, we provide evidence that pomegranate consumption led to a shift in distal gut microbiota towards a more beneficial state in the majority of subjects, and such shifts were statistically associated with the increased levels of total urinary polyphenols. The results further add to the available scientific evidence that pomegranate eating is associated with beneficial effects. In regions with populations with known gut health problems and abundant pomegranate production, such as Africa and the Middle East, incorporation of this fruit into regular diet may improve intestinal health. Further studies are warranted on larger sample sizes and with different dosages in health and in disease states.

## Data Availability

The original contributions presented in this study are included in the article. Further inquiries can be directed to the corresponding authors.
